# Azygos vein varix mimicking bronchial cysts

**DOI:** 10.1002/rcr2.353

**Published:** 2018-07-18

**Authors:** Hiroyuki Miura, Jun Miura, Hiroshi Hirano

**Affiliations:** ^1^ Department of Thoracic Surgery Akiru Municipal Medical Center Akiruno Tokyo Japan; ^2^ Department of Surgery Kyorin University School of Medicine Mitaka Tokyo Japan; ^3^ Department of Pathology Hachioji Medical Center of Tokyo Medical University Hachioji Tokyo Japan

**Keywords:** Azygos vein, azygos vein aneurysm, azygos vein varix, mediastinal cyst, mediastinal tumour

## Abstract

Azygos vein varix is a very rare disease associated with a risk of rupture or thrombosis. Here, we report a case of azygos vein varix mimicking bronchogenic cysts. A 79‐year‐old female patient presented with abnormal shadows on a chest X‐ray during an annual check‐up. A plain chest computed tomography (CT) showed a well‐defined tumour, approximately 66 X 65 X 45 mm in size, behind the trachea. An enhanced CT scan was refused due to asthma complications. A high signal on T2W1 images from magnetic resonance imaging (MRI) indicated a cystic tumour. Diagnosed as a bronchogenic cyst, a cyst excision was scheduled. Upon observation with a thoracoscope, a dark purple‐red saccular tumour was observed continuing on to the azygos vein and was removed using linear staplers. The patient is alive and well 4 years after the operation. If a cystic tumour located in the right upper mediastinum is observed, the presence of azygos vein varix should be considered.

## Introduction

Azygos vein varix is a very rare disease and is mostly asymptomatic. Most cases are detected on chest X‐rays by chance. The disease is operable, and surgery is often performed to avoid rupture or thrombosis. Contrast‐enhanced computed tomography (CT) scans or enhanced magnetic resonance imaging (MRI) scans are useful for the diagnosis. However, in instances where contrast agents are refused due to complications such as asthma, azygos vein varix resembles a mediastinal tumour similar to bronchogenic cysts.

## Case Report

**Figure 1:**
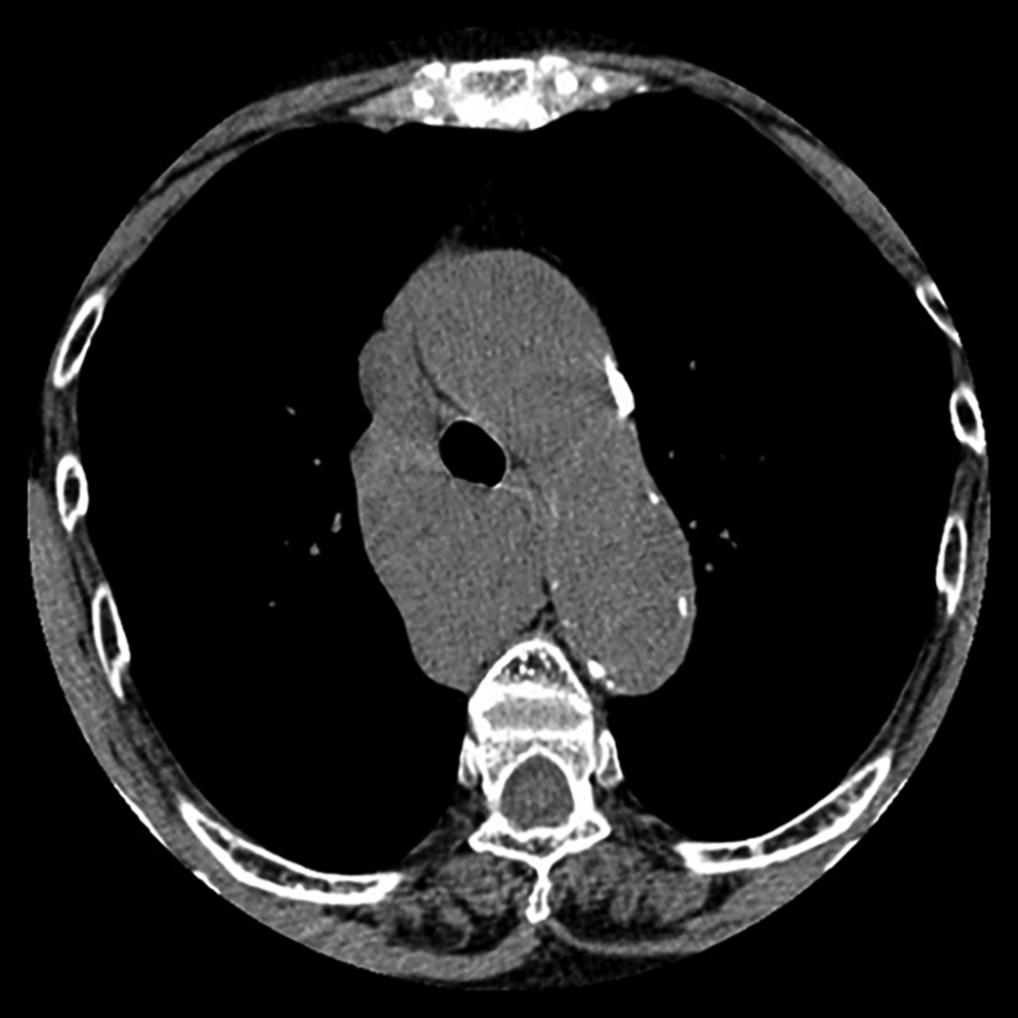
Computed tomography (CT) showed a well‐defined tumour behind the trachea.

**Figure 2:**
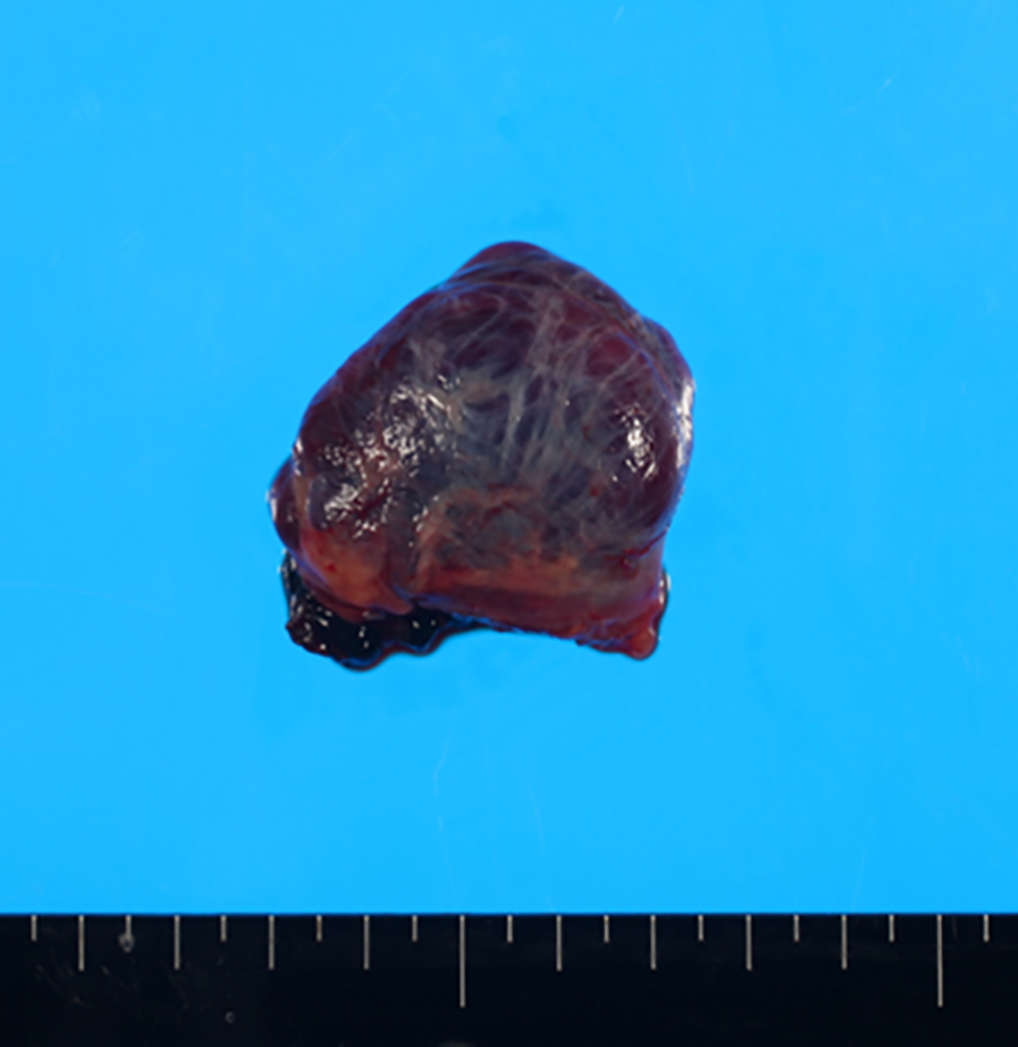
A dark purple‐red saccular tumour was observed continuing on to the azygos vein.

A 79‐year‐old female patient presented with abnormal shadows on a chest X‐ray during an annual check‐up. The patient had a history of asthma and skin cancer. There was no history of trauma. Blood tests, including for haematology, renal, and hepatic function, were within normal ranges. There were no abnormal findings on electrocardiograms. A plain chest CT showed a well‐defined tumour approximately 66 X 65 X 45 mm in size behind the trachea. The tumour was homogenous with 45 Hounsfield units (HU) (Fig. 1). Calcification or fat components were not found. Bronchogenic cyst, neurogenic tumour, Castleman disease, and malignant lymphoma were considered possible definitive diagnoses. A high signal on T2W1 images from an MRI indicated a cystic tumour. After explaining the side effects of the contrast agent in patients with asthma, the patient refused an enhanced CT scan. Diagnosed as a bronchogenic cyst, a cyst excision was scheduled. Upon observation with a thoracoscope, a dark purple‐red saccular tumour was observed continuing on to the azygos vein (Fig. 2). The top of the tumour adhered to the superior vena cava (SVC). The peripheral and proximal sites of the varix were excised using a linear stapler, and the tumour was removed. While waiting for the normalization of liver function, the patient was discharged 6 days after the operation. The patient is alive and well 4 years after the operation.

## Discussion

Azygos vein varix is very rare, and most of the tumours are detected during annual check‐ups without symptoms. Although cases with heart failure, portal hypertension, and obstruction of the inferior vena cava have been reported, considering its occurrence in a young patient 1, the cause of the disease is suspected to be the congenital weakness of the azygos vein. Kurihara et al. 2 hypothesized that a remnant of the foetal vein, such as the right posterior cardinal vein, subcardinal vein or primitive subclavian veins, may be the origin of a saccular aneurysm of the azygos vein. Therefore, the term diverticulum of the azygos vein is considered an appropriate term for azygos vein aneurysms. Many authors have reported this type of tumour as an azygos vein aneurysm. However, because the azygous vein is a vein, varix is a more appropriate term than aneurysm.

Contrast‐enhanced CT and enhanced MRI scans are useful for the diagnostic procedure. However, our patient suffered from asthma. Therefore, enhanced CT and enhanced MRI scans could not be performed. Without the enhanced imaging, the tumour resembled bronchogenic cysts or foregut duplication cysts. Previous reports have shown that preoperative diagnoses include bronchogenic cysts, neurogenic tumours, and swollen lymph nodes 2, 3, 4.

A previous report describes a case where the tumour did not change in size for 4 years, and surgery was not performed 3. However, as long as cases with ruptures and/or thrombosis 1, 4, 5 are reported, the surgical removal of the tumour should be considered the best approach for this type of tumour. Successful video‐assisted thoracic surgeries for this tumour have been reported. If there is a thrombus in the tumour, there is a risk of thrombus inflow floating into the heart through the SVC. There is a need to ligate the SVC side of the tumour in advance. If this technique cannot be performed, a mini thoracotomy should promptly be performed to avoid thrombosis.

If cystic tumours located in the right upper mediastinum are observed, the presence of azygos vein varix should be considered.

### Disclosure Statement

Appropriate written informed consent was obtained for publication of this case report and accompanying images.
